# Brassica database (BRAD) version 2.0: integrating and mining Brassicaceae species genomic resources

**DOI:** 10.1093/database/bav093

**Published:** 2015-11-20

**Authors:** Xiaobo Wang, Jian Wu, Jianli Liang, Feng Cheng, Xiaowu Wang

**Affiliations:** Institute of Vegetables and Flowers, Chinese Academy of Agricultural Sciences, Beijing 100081, China

## Abstract

The Brassica database (BRAD) was built initially to assist users apply *Brassica rapa *and *Arabidopsis thaliana* genomic data efficiently to their research. However, many Brassicaceae genomes have been sequenced and released after its construction. These genomes are rich resources for comparative genomics, gene annotation and functional evolutionary studies of *Brassica *crops. Therefore, we have updated BRAD to version 2.0 (V2.0). In BRAD V2.0, 11 more Brassicaceae genomes have been integrated into the database, namely those of *Arabidopsis lyrata*,* Aethionema arabicum*,* Brassica oleracea*,* Brassica napus*,* Camelina sativa*, *Capsella rubella*,* Leavenworthia alabamica*, *Sisymbrium irio* and three extremophiles *Schrenkiella parvula*,* Thellungiella halophila *and* Thellungiella salsuginea*. BRAD V2.0 provides plots of syntenic genomic fragments between pairs of Brassicaceae species, from the level of chromosomes to genomic blocks. The Generic Synteny Browser (GBrowse_syn), a module of the Genome Browser (GBrowse), is used to show syntenic relationships between multiple genomes. Search functions for retrieving syntenic and non-syntenic orthologs, as well as their annotation and sequences are also provided. Furthermore, genome and annotation information have been imported into GBrowse so that all functional elements can be visualized in one frame. We plan to continually update BRAD by integrating more Brassicaceae genomes into the database.

**Database URL:** http://brassicadb.org/brad/

## Introduction

Brassicaceae is a large eudicot family that includes the model plant *Arabidopsis thaliana*. The Brassicaceae family has a remarkable diversity of species, genetics and morphotypes, as well as scientific and economic importance. Brassicaceae species have become model systems for studies of polyploidy and evolution (1). The important genus *Brassica* of Brassicaceae contains many vegetable, condiment and oil species that account for about 12% of the world’s edible vegetable oil production (http://faostat.fao.org/). U’s triangle theory (2) has been applied to describe the relationships among six widely cultivated *Brassica* species, the diploids *Brassica rapa* (AA), *B. nigra* (BB) and *B. oleracea* (CC) and their allotetraploids *B. juncea* (AABB), *B. napus* (AACC) and *B. carinata* (BBCC). Of these, the *B. rapa* genome was the first to be sequenced in 2011 (3) and the original *Brassica *database was built based on it (4).

BRAD version 1.0 (V1.0) provides *B. rapa* genome sequences and gene models, as well as all the syntenic and non-syntenic homologous gene pairs between *B. rapa *and *A. thaliana*. On all its pages, BRAD V1.0 incorporates a useful navigation dialog-window that provides links to every* B. rapa* and *A. thaliana* gene ID. The small navigation window directs users by integrating relevant resource links of the target gene. With the rapid development of next-generation sequencing technology and the dramatic decrease in cost, many Brassicaceae species have been sequenced or were planned to be sequenced after BRAD V1.0 was constructed. Recently, the genomes of *B. rapa* sister species, *B. oleracea *and *B. napus*, have been sequenced (5, 6) and nine other Brassicaceae species have also been sequenced (7–13). These 13 Brassicaceae genome datasets are a valuable resource for genome and gene studies among the closely related Brassicaceae species.

To help researchers and breeders use these recently released Brassicaceae genome sequences efficiently in scientific investigations and breeding applications, we have updated BRAD to version 2.0 (V2.0). BRAD V2.0 contains updated datasets and functions that include all syntenic gene pairs between *A. thaliana *and the other Brassicaceae species, more genome and gene sequences and gene annotations, as well as syntenic figures and genome visualization of all the incorporated Brassicaceae species in the Genome Browser (GBrowse) (14). BRAD V2.0 provides a comprehensive framework for comparative genomic analysis and studies of the evolution of gene function across Brassicaceae species, especially for the *Brassica *crops.

## BRAD V2.0: feature updates

### Overview of BRAD V2.0

In BRAD V1.0, datasets of genome and gene sequences, gene annotations, non-coding RNAs, transposable elements, genetic markers and linkage maps of *B. rapa* were provided (15, 16). A navigation dialog-window for every gene of *B. rapa* and *A. thaliana* was provided to help users obtain all related information. Furthermore, BLAST and GBrowse tools (16) were embedded in BRAD for sequence alignment and for visualizing genomic elements, respectively.

BRAD V1.0 has now been updated to V2.0 to include Brassicaceae genome sequences that have been released recently. In BRAD V2.0, a new section has been incorporated that shows genomic synteny and micro-fragmental synteny between any two Brassicaceae species. An alternative pairwise synteny plotting tool, the Generic Synteny Browser (GBrowse_syn) module (17) of GBrowse, has been included to visualize local synteny relationships among multiple genomes. Moreover, genome and gene sequences, gene annotations and syntenic and non-syntenic orthologs between *A. thaliana* and other Brassicaceae species have been integrated into different sections of BRAD V2.0.

### Technical details

All genomic data were processed using the tool SynOrths tool (15) to generate genome and gene level synteny datasets. Then, syntenic figures were generated based on these synteny datasets and stored in a MySQL (18) database.

Genome sequences, gene models and the processed datasets, including all syntenic genes, gene annotation information and specific gene families were all imported into MySQL, which enables multifaceted browsing and searching in BRAD. Furthermore, a standalone BLAST (19) service implemented in BRAD allows sequence searches against Brassicaceae genomes, protein-coding gene sequences and protein sequences. The GBrowse package, which is commonly used to visualize genomic datasets, remains in BRAD V2.0 to view bulk genomic elements of the Brassicaceae species. Furthermore, the syntenic datasets are provided not only as tabular results and pairwise-genome synteny images in the keyword search section, but also are visualized as a multiple genome synteny comparison in the GBrowse module GBrowse_syn.

### BRAD stocks: Brassicaceae genomes

Statistics of the Brassicaceae genomic data, including genome sequences, predicted gene models, protein-coding gene sequences and protein sequences are shown in Table 1. In total, about 4 Gb of data have been collected in BRAD V2.0. In addition to the original genome sequences and gene models, seven types of annotation for the predicted genes have been generated. The annotations have been sourced from the Swiss-Prot, TrEMBL (20), KEGG (Kyoto Encyclopedia of Genes and Genomes) (21), InterPro (22) and Gene Ontology (GO) (23) databases and syntenic genes and BLASTX alignments (best hit, e-value 1E-05) of Brassicaceae genes to the *A. thaliana* genome also have been included. The numbers of annotation records in these datasets for these species (excluding *A. thaliana*) are shown in Table 2. We used InterProScan (V48.0) (24), which includes 28 175 GO terms, to generate the InterPro domain and GO annotations. When InterProScan is updated, the GO annotations also will be updated in BRAD.

**Table 1.** Overview of the 13 Brassicaceae genomes in BRAD V2.0

Species	Genome size (Mb)	No. of chromosomes	No. of genes	Status	Source
*A. thaliana*	120	5	27 416	Chromosome	TAIR (https://www.arabidopsis.org/)
*A. lyrata*	207	8	32 670	Chromosome	(http://www.phytozome.net/)
*A. arabicum*	203	11	37 839	Scaffold	(http://mustang.biol.mcgill.ca:8885/)^a^
*B. rapa*	284	10	41 174	Chromosome	BRAD (http://brassicadb.org/brad/)
*B. oleracea*	540	9	45 758	Chromosome	BolBase (http://ocri-genomics.org/bolbase/index.html)^a^
*B. napus*	840	19	101 040	Chromosome	CoGe (https://genomevolution.org/CoGe/)^a^
*C. sativa*	641	20	94 495	Chromosome	(http://www.camelinadb.ca)
*C. rubella*	135	8	28 447	Chromosome	(http://www.phytozome.net/)
*L. alabamica*	174	11	38 676	Scaffold	(http://mustang.biol.mcgill.ca:8885/)^a^
*S. irio*	259	7	49 956	Scaffold	(http://mustang.biol.mcgill.ca:8885/)^a^
*S. parvula*	114	7	28 901	Chromosome	GenBank (http://www.ncbi.nlm.nih.gov/genbank)
*T. halophila*	243	7	29 284	Scaffold	(http://www.phytozome.net/)
*T. salsuginea*	234	7	28 457	Scaffold	GenBank (http://www.ncbi.nlm.nih.gov/genbank)

^a^Collaboration with project investigator for genome analysis*.*

**Table 2.** Numbers of annotation records in BRAD V2.0 from the Gene Ontology (GO), InterPro, KEGG, Swiss-Prot and TrEMBL databases, as well as syntenic genes (Orthologs) and BLASTX alignments (best hits) to the* A. thaliana* genome sequence

Species	GO	InterPro	KEGG	Swiss-Prot	TrEMBL	Orthologs	BLASTX
*A. arabicum*	32 609	56 964	28 773	21 689	20 342	15 754	26 910
*A. lyrata*	53 457	64 268	29 723	22 396	31 780	22 552	32 524
*B. rapa*	62 875	62 852	20 463	28 501	37 220	26 194	40 946
*B. oleracea*	76 109	85 261	21 071	30 504	40 498	31 794	45 603
*B. napus*	96 202	136 419	88 173	68 590	44 436	59 707	89 257
*C. rubella*	38 109	59 533	27 927	22 123	26 195	20 952	27 973
*C. sativa*	112 461	168 977	90 572	71 615	35 161	64 433	91 080
*L. alabamica*	42 588	66 021	33 062	25 932	22 838	24 259	32 277
*S. irio*	38 116	67 961	35 969	26 150	24 992	18 224	33 522
*S. parvula*	36 542	53 883	25 986	20 179	21 721	20 846	28 827
*T. halophila*	39 795	61 350	28 634	22 732	23 398	19 819	28 594
*T. salsuginea*	33 820	52 397	25 730	20 158	23 104	19 328	25 903
Total	662 683	935 886	456 083	380 569	351 685	343 862	503 416

### Updated feature: genome synteny analysis

Genome synteny analysis provides information for studies into the evolution of genome and gene function among species. BRAD V1.0 provided syntenic gene pairs between *B. rapa* and *A. thaliana* so that the gene information of the well-studied model plant *A. thaliana* could be used to annotate *B. rapa* genes*.* In BRAD V2.0, whole-genome synteny relationships between *A. thaliana* genes and the genes of other Brassicaceae species have been generated and integrated. We obtained syntenic gene pairs that ranged from 17 800 between *A. thaliana* and *Aethionema arabicum* to 59 191 between *A. thaliana* and *Camelina sativa *(Table 3 and Supplementary Tables S1 and S2). The number of tandem gene arrays is shown in Table 4; most had syntenic counterparts in the *A. thaliana* genome. These datasets can be used to investigate genomic rearrangement history, share gene annotation information and investigate functional differentiation of orthologous genes among Brassicaceae species.

**Table 3.** Numbers of syntenic genes in 24 genomic blocks of the three subgenomes in *B. rapa* and *B. oleracea. *The three subgenomes were partitioned as described previously (3), and named as least fractionated subgenome (LF), more fractionated subgenome 1 (MF1) and more fractionated subgenome 2 (MF2)

Genomic blocks	*B. rapa*			*B. oleracea*		
	LF	MF1	MF2	LF	MF1	MF2
A	1230	653	785	1163	645	756
B	796	500	495	746	483	478
C	422	324	257	394	333	256
D	63	209	283	43	205	257
E	980	687	492	953	650	487
F	1567	1098	898	1483	1044	873
G	35	17	78	37	16	71
H	263	167	171	248	166	166
I	366	369	82	343	339	83
J	1152	926	726	1120	881	698
K	141	128	87	146	117	76
L	247	184	120	232	171	114
M	276	135	125	277	126	105
N	811	538	450	761	526	440
O	294	168	81	278	173	82
P	150	124	66	138	121	54
Q	307	179	178	299	173	184
R	1303	879	866	1274	836	824
S	259	129	69	274	125	63
T	96	110	109	88	110	109
U	1636	1082	821	1582	1028	791
V	280	197	222	276	169	209
Wa	111	77	52	104	67	51
Wb	619	464	501	574	428	446
X	489	242	270	481	234	271
Total	13 893	9586	8284	13 314	9166	7944

**Table 4.** Tandem array statistics for each Brassicaceae species in BRAD V2.0

Species	Tandem (arrays|genes)	Syntenic tandem (arrays|genes)	Ratio (syntenic tandem/tandem) (%)
*B. rapa*	2041|4896	1570|3796	76.9
*B. oleracea*	1823|4223	1290|2960	70.8
*S. parvula*	1139|2700	1022|2545	89.7
*A. lyrata*	1751|4388	1441|3743	82.3
*L. alabamica*	789|1769	454|1026	57.5
*C. rubella*	1619|4377	1397|3691	86.3
*S. irio*	1760|4221	1080|2710	61.4
*A. arabicum*	1355|3557	880|2377	64.9
*T. halophila*	1414|3642	990|2491	70.0
*T. salsuginea*	1401|3378	975|2337	69.6
*B. napus*	4406|10 228	2317|5355	52.6
*C. sativa*	5713|13 961	1121|2722	19.6

*Brassica* crops experienced a common and relatively recent (9–15 million years ago) whole-genome triplication event after three rounds of polyploidization (γ, β and α whole-genome duplication) in Brassicaceae (3, 5, 6, 8, 25). They have three subgenomes in their genomes compared with other Brassicaceae species. *B. napus* is the allotetraploid of *B. rapa* and* B. oleracea*, thus its genome is composed of six subgenomes. Additionally, *C. sativa* experienced an independent and more recent whole-genome triplication event than the event in *Brassica*. Based on the rules that have been used to partition the three subgenomes of *B. rapa* (3, 26), syntenic paralogous genes in the subgenomes of the four polyploidy species mentioned above were separated and updated in BRAD V2.0.

Syntenic gene pairs were plotted as dots on a two-dimensional figure, where the x and y axes denote the chromosomal positions of the genes in any two genomes. Continuously distributed syntenic genes in any two genomes generate dot plots with fragments of lines (Figure 2B). The dot-formed lines that are produced represent the chromosomal fragments and their different arrangements between two genomes. The ancestral genomic blocks (GBs) (27, 28) of corresponding chromosomal fragments are also shown (Figure 2B).

## Genome synteny resource guidelines

### Mining syntenic genes

**Figure 1.**
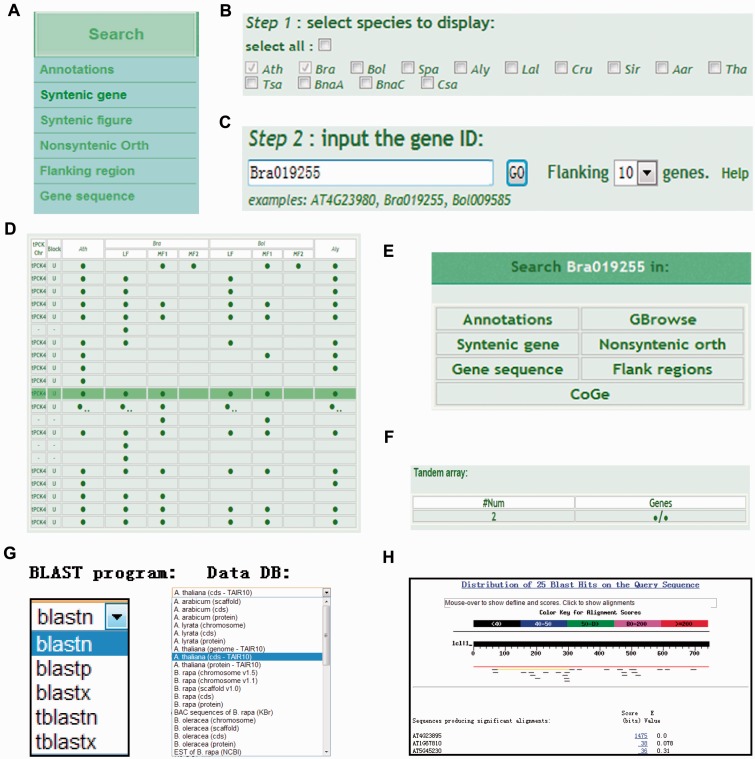
Using the ‘Syntenic gene’ search option in BRAD V2.0. (**A**) The ‘Syntenic gene’ option is selected by placing the cursor over the Search section and clicking on ‘Syntenic gene’. (**B**) Select the Brassicaceae species genomes to be searched using the checkboxes. (**C**) Input the ID of the gene to be searched. (**D**) The results of the syntenic gene search in the selected species are output in a table. The solid circles represent genes. Clicking on a circle opens a dialog-window. Clicking on a tandem array symbol (‘two small dots’) outputs the tandem array. (**E**) The dialog-window provides navigation of links to information about the target gene. (**F**) The output tandem array is displayed under the results table. (**G**) The BLAST services can be selected from the Tools section. (**H**) BLAST alignments of nucleotide protein-coding sequences (CDS) of *A. thaliana*.

BRAD V2.0 has five main sections: Browse, Search, Tools, Download and Links. Placing the cursor over the Search section activates a drop-down menu. Clicking on the ‘Syntenic gene’ option (Figure 1A) opens the search syntenic genes page where checkboxes for 11 Brassicaceae species (*B. napus* contains the *Brassica* A and C subgenomes) allow users to choose their required searches; a syntenic gene search between* A. thaliana* and *B. rapa* is set as the default (Figure 1B). Next, users are required to provide a gene ID to search for syntenic genes among the selected species (Figure 1C). The number of genes flanking the syntenic genes can be selected from a drop-down list as 10, 20 or 50. The search is activated by clicking the ‘GO’ button. For example, by selecting *B. oleracea* and *A. lyrata* as the species, inputting Bra019255 as the gene ID , setting the number of flanking genes to 10 (the default) and clicking the GO button, the results are output in a table that appears below the search panel as shown in Figure 1D. The solid circles indicate genes. Information about a gene can be obtained by placing the cursor over a circle. Clicking on the solid circle opens a pop-up dialog-window in which navigation information for the target gene is displayed (Figure 1E). Clicking on a tandem symbol (two small dots following a gene symbol) displays the corresponding tandem gene array information at the bottom of the search page (Figure 1F).

**Figure 2.**
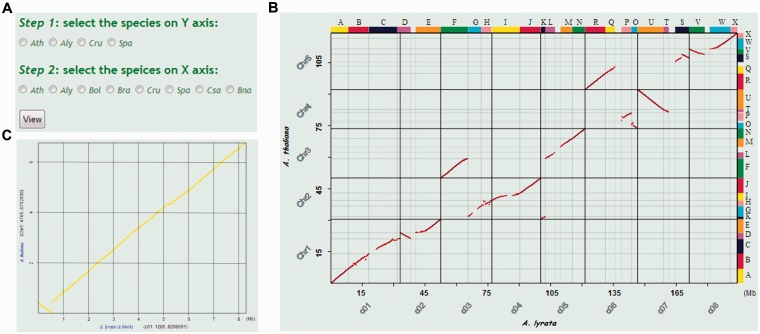
Obtaining pairwise syntenic dot plots between two Brassicaceae genomes in BRAD V2.0. The ‘Syntenic figure’ option is selected by placing the cursor over the Search section and clicking on ‘Syntenic figure’. (**A**) Select the Brassicaceae species genomes to be plotted. (**B**) Syntenic figure between *A. thaliana* and *A. lyrata*. Syntenic gene pairs are plotted as red dots. Genomic blocks are shown as color-coded bars labeled A–X on the top and right sides of the plot. The genome sequences of *A. lyrata* and* A. thaliana* are displayed on the x and y axes, respectively. (**C**) Syntenic figure of local genomic block A obtained by clicking on the corresponding block. Chromosome labels and the intervals of the A block are given in brackets after the labels on the two axes.

Users can also input their own nucleotide sequences instead of gene IDs using the BLAST services (Blastn, Blastp, Blastx tBlastn and tBlastx) provided under the Tools section in BRAD V2.0. The BLAST search page allows users to search against bulk data from different Brassicaceae sequence databases such as genomes, BACs, protein-coding genes, proteins and ESTs (Figure 1G). Users will obtain related gene IDs based on the BLAST alignments as output (Figure 1H). The obtained gene IDs can be used as input for the search syntenic genes analysis described above (Figure 1B–F). Furthermore, if a user’s nucleotide sequences are not derived from gene regions, the user may still be able to obtain the location of their sequences in the genomes of certain species. This information can be used to retrieve the flanking sequences and elements, which can be visualized or downloaded from GBrowse under the Tools section in BRAD V2.0.

### Visualization of synteny analysis

A new ‘Syntenic figure’ function is available under the Search section in BRAD V2.0, which can be used to better illustrate the genomic synteny relationship between two Brassicaceae species. This function can be used to plot genomic synteny relationships as two-dimensional figures. One of the four ancestral species (*A. thaliana*, *A. lyrata*, *C. rubella* and *S. parvula*) can be selected for display on the y axis and one of eight other Brassicaceae species can be selected for display on the x axis by clicking the corresponding checkboxes. A total of 28 such figures are available (ignoring self-to-self plots). For example, if ‘Ath’ is chosen for the y axis and ‘Aly’ is chosen for the x axis, then by clicking the ‘View’ button (Figure 2A), users will obtain the image shown in Figure 2B. The lines formed by the red dots show the genomic synteny relationships between the two genome sequences. Clicking on any of the GB regions (shown in color-coded bars), such as GB ‘A’, opens a figure that shows detailed synteny information (Figure 2C). Clicking a dot, which represents a particular gene, on the GB figure will open the GBrowse_syn Web page (17) and show the 100-Kb genomic region flanking the clicked dot.

### Syntenic blocks analysis for multiple genome resources

**Figure 3.**
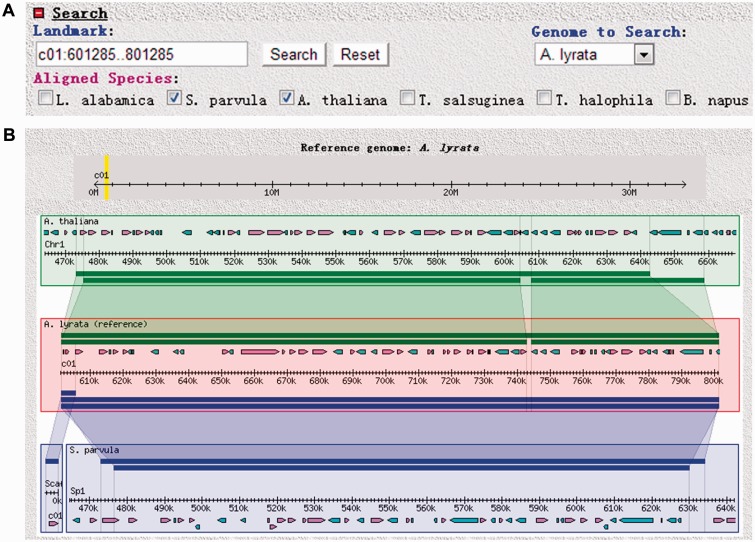
Using GBrowse_syn for local syntenic visualization of Brassicaceae genome sequences in BRAD V2.0**.** The ‘GBrowse_syn’ option is selected by placing the cursor over the Tools section and clicking on ‘GBrowse_syn’. (**A**) Search panel for GBrowse_syn. (**B**) Representative output showing local synteny relations among the *A. thaliana*, *S. parvula* and *A. lyrata* genomes.

The GBrowse_syn (16) tool for visualizing synteny or collinear genomic regions among multiple genomes can be accessed from the Tools section of BRAD 2.0. GBrowse_syn uses species name and genomic position consisting of the chromosome label, and start and stop positions as input. For example, if ‘c01:601,285..801,285’ is input in the Landmark search box and *A. lyrata* is selected as the target species from the Genome to Search drop-down list, then the genomic region from 601 285 bp to 801 285 bp on chromosome 1 of *A. lyrata *will be searched (Figure 3A). By checking the boxes of *A. thaliana* and *S. parvula *(Figure 3A) and clicking the ‘Search’ button next to the Landmark search box, a visualization of syntenic blocks for the multiple genomes is obtained (Figure 3B). The sequence of the target species (in this case *A. lyrata*) is shown in the middle of the graph as the reference genome, and the genomes being compared with the reference are displayed above and below it. Clicking on the track of a compared species changes it into the reference species and all others become the compared genomes. Furthermore, a link to the ‘Syntenic gene’ search section is provided for each gene icon shown on the graph of multiple genome syntenies.

## Discussion and conclusions

Many *Brassica* databases have been built to better understand and use the genomic datasets from *Brassica* species. These databases include the Brassica Database BRAD (http://brassicadb.org), Brassica.info (http://www.brassica.info/), BrassEnsembl (http://www.brassica.info/BrassEnsembl/index.html), BrassicaDB (http://brassica.nbi.ac.uk/BrassicaDB/), CropStoreDB (http://www.cropstor edb.org/) and BolBase (http://www.ocri-genomics.org/bol base/index.html). These databases all have different emphasis. Brassica.info integrates information about genomic resources and releases news of projects or activities on Brassica studies. It also provides downloading services for some genome data. BrassEnsembl visualizes different sets of Brassica genomic data under a single frame. CropStoreDB provides a practical approach to managing crop genetic data, whereas BolBase (*B. oleracea* Genome Database) is focused on genomic structure comparisons of the *B. oleracea* genome. Unlike these other databases, BRAD uses information from genomic studies and gene function studies in the model species *A. thaliana* to annotate the newly sequenced genomes of* Brassica* species.

BRAD V2.0 is a substantially improved version of BRAD V1.0. In BRAD V2.9, more Brassicaceae genomes have been integrated, and comprehensive functional annotations of all the Brassicaceae gene models, genome and gene-level syntenic datasets and visualization tools have been provided. In addition, we have included a new application ‘Syntenic figure’ in the search section to allow users to view pairwise syntenic relationships between the Brassicaceae genomes in BRAD V2.0. We used the GBrowse_syn module to visualize multiple genome synteny. The inclusion of bulk Brassicaceae genome datasets and new applications make BRAD V2.0 a user friendly platform from which to conveniently retrieve genomic information from the genome to gene levels. The updated BRAD V2.0 will be a valuable resource for research into comparative genomics, plant evolution and molecular biology, as well as for breeders of Brassicaceae crops.

## Funding

973 program (2012CB113900 and no. 2013CB127000), the 863 program (2012AA100101), the National Natural Science Foundation of China (grant no. 31301771) and the Science and Technology Innovation Program of the Chinese Academy of Agricultural Sciences. Research was carried out in the Key Laboratory of Biology and Genetic Improvement of Horticultural Crops, Ministry of Agriculture, China.

*Conflict of interest*. None declared.

## Supplementary Data

Supplementary data are available at *Database* Online.

Supplementary Data
