# Supplementary material for: Brassica database (BRAD) version 2.0: integrating and mining Brassicaceae species genomic resources
Source: Database (Oxford). 2015 Nov 20;2015:bav093. doi: 10.1093/database/bav093 (PMC4653866; doi:10.1093/database/bav093)
Supplement: Supplementary Data [file supp_2015_bav093_index.html]

Supplementary Data 

# Brassica database (BRAD) version 2.0: integrating and mining Brassicaceae species genomic resources

## Supplementary Data

files

- Supplementary Data - docx file
