# Supplementary material for: Brassica database (BRAD) version 2.0: integrating and mining Brassicaceae species genomic resources
Source: Database (Oxford). 2015 Nov 20;2015:bav093. doi: 10.1093/database/bav093 (PMC4653866; doi:10.1093/database/bav093)
Supplement: Supplementary Data [file supp_bav093_supplementary_tables.docx]

**Supplementary Tables**

**Table S1.** Number of syntenic genes in the 24 GBs from eight Brassicaceae species*.*

| **Genomic**  **block** | ***S.parvula*** | ***A.lyrata*** | ***L.alabamica*** | ***C.rubella*** | ***S.irio*** | ***A.arabicum*** | ***T.halophila*** | ***T.salsuginea*** |
| --- | --- | --- | --- | --- | --- | --- | --- | --- |
| **A** | 1,572 | 1,822 | 1,575 | 1,735 | 1,556 | 1,330 | 1,608 | 1,556 |
| **B** | 1,137 | 1,329 | 1,027 | 1,298 | 989 | 993 | 1,136 | 1,088 |
| **C** | 713 | 906 | 686 | 903 | 619 | 624 | 774 | 627 |
| **D** | 522 | 647 | 425 | 637 | 392 | 457 | 501 | 466 |
| **E** | 1,390 | 1,522 | 1271 | 1,529 | 1,191 | 1,201 | 1,358 | 1,312 |
| **F** | 2,142 | 2,428 | 2,009 | 2,311 | 2,035 | 2,008 | 2,136 | 2,105 |
| **G** | 113 | 197 | 115 | 178 | 47 | 60 | 120 | 100 |
| **H** | 430 | 503 | 383 | 516 | 385 | 395 | 439 | 418 |
| **I** | 614 | 732 | 549 | 718 | 436 | 478 | 611 | 521 |
| **J** | 1,657 | 1,878 | 1,589 | 1,826 | 1,581 | 1,537 | 1,665 | 1,648 |
| **K** | 213 | 253 | 204 | 261 | 201 | 217 | 219 | 212 |
| **L** | 346 | 396 | 272 | 404 | 294 | 364 | 311 | 311 |
| **M** | 454 | 629 | 411 | 635 | 357 | 506 | 478 | 450 |
| **N** | 1,072 | 1,228 | 1,010 | 1,193 | 1,055 | 887 | 1,102 | 1,078 |
| **O** | 404 | 437 | 356 | 472 | 377 | 379 | 398 | 404 |
| **P** | 286 | 376 | 239 | 385 | 184 | 257 | 306 | 263 |
| **Q** | 364 | 467 | 356 | 466 | 372 | 336 | 388 | 387 |
| **R** | 1,704 | 2,004 | 1,751 | 1,957 | 1,730 | 1,589 | 1,825 | 1,809 |
| **S** | 410 | 545 | 398 | 570 | 310 | 329 | 403 | 381 |
| **T** | 292 | 358 | 270 | 370 | 205 | 255 | 308 | 266 |
| **U** | 2,120 | 2,324 | 1,958 | 2,264 | 2,007 | 1,709 | 2,140 | 2,043 |
| **V** | 437 | 569 | 417 | 540 | 363 | 412 | 474 | 445 |
| **Wa** | 131 | 191 | 127 | 172 | 141 | 55 | 145 | 143 |
| **Wb** | 941 | 1,070 | 913 | 1,052 | 821 | 842 | 975 | 915 |
| **X** | 615 | 695 | 612 | 662 | 610 | 580 | 617 | 623 |
| **Total** | 20,079 | 23,506 | 18,923 | 23,054 | 18,258 | 17,800 | 20,437 | 19,571 |

**Table S2.** Number of syntenic genes in the 24 GBs from each subgenome in *B. napus* and *C. sativa.*

| **Genomic**  **block** | ***B. napus (A)*** | | | ***B. napus (C)*** | | | ***C. sativa*** | | |
| --- | --- | --- | --- | --- | --- | --- | --- | --- | --- |
|  | **LF** | **MF1** | **MF2** | **LF** | **MF1** | **MF2** | **LF** | **MF1** | **MF2** |
| **A** | 978 | 522 | 466 | 916 | 479 | 551 | 1,607 | 1,572 | 1,598 |
| **B** | 593 | 382 | 293 | 473 | 304 | 319 | 1,057 | 1,087 | 1,084 |
| **C** | 289 | 244 | 166 | 250 | 206 | 131 | 746 | 750 | 744 |
| **D** | 8 | 117 | 202 | 20 | 125 | 160 | 470 | 476 | 480 |
| **E** | 749 | 473 | 382 | 785 | 346 | 353 | 1,300 | 1,301 | 1,301 |
| **F** | 1,229 | 617 | 709 | 1,120 | 504 | 671 | 2,061 | 2,105 | 2,037 |
| **G** | 29 | 17 | 67 | 3 | 17 | 57 | 147 | 143 | 142 |
| **H** | 186 | 111 | 132 | 139 | 98 | 117 | 416 | 430 | 405 |
| **I** | 298 | 292 | 70 | 289 | 275 | 58 | 613 | 610 | 613 |
| **J** | 839 | 620 | 572 | 728 | 592 | 538 | 1,607 | 1,612 | 1,615 |
| **K** | 106 | 91 | 55 | 107 | 76 | 50 | 204 | 207 | 195 |
| **L** | 189 | 131 | 81 | 170 | 120 | 80 | 303 | 295 | 304 |
| **M** | 164 | 69 | 100 | 149 | 64 | 76 | 458 | 472 | 458 |
| **N** | 606 | 393 | 337 | 665 | 339 | 301 | 1,073 | 1,068 | 1,065 |
| **O** | 175 | 135 | 50 | 112 | 128 | 44 | 361 | 378 | 377 |
| **P** | 102 | 92 | 51 | 82 | 86 | 20 | 291 | 281 | 287 |
| **Q** | 248 | 121 | 148 | 218 | 104 | 157 | 378 | 387 | 386 |
| **R** | 1,071 | 626 | 443 | 900 | 705 | 566 | 1,768 | 1,761 | 1,778 |
| **S** | 193 | 105 | 36 | 153 | 93 | 39 | 418 | 426 | 407 |
| **T** | 62 | 88 | 67 | 62 | 53 | 47 | 278 | 302 | 299 |
| **U** | 1,228 | 730 | 564 | 1,226 | 817 | 514 | 2,018 | 2,014 | 1,998 |
| **V** | 149 | 137 | 142 | 192 | 100 | 140 | 447 | 449 | 440 |
| **Wa** | 98 | 53 | 45 | 84 | 56 | 35 | 146 | 143 | 151 |
| **Wb** | 417 | 334 | 344 | 344 | 323 | 289 | 927 | 916 | 925 |
| **X** | 375 | 116 | 206 | 316 | 131 | 209 | 604 | 606 | 613 |
| **Total** | 10,381 | 6,616 | 5,728 | 9,503 | 6,141 | 5,522 | 19,698 | 19,791 | 19,702 |
